# Renal Cell Carcinoma With Tumor Thrombus: A Case Series in Puerto Rico

**DOI:** 10.7759/cureus.58113

**Published:** 2024-04-12

**Authors:** Sebastián A Bernaschina-Rivera, Gustavo Alayón-Rosario, Gabriel Dieppa-Barnés, Jarline Encarnación, Carmen Ortiz-Sánchez, Rafael Santini-Domínguez, Jorge L Martínez-Trabal, Gilberto Ruiz-Deyá

**Affiliations:** 1 Medicine, Ponce Health Sciences University, Ponce, PRI; 2 Medicine, University of Puerto Rico, Mayagüez, PRI; 3 General Surgery, St Luke's Episcopal Medical Center, Ponce, PRI; 4 Surgery, Ponce Health Sciences University, Ponce, PRI; 5 Urology, St Luke's Episcopal Medical Center, Ponce, PRI

**Keywords:** intravascular extension, tumor thrombus, renal cell carcinoma, puerto rico, laparoscopic nephrectomy

## Abstract

Introduction: Renal cell carcinoma (RCC) is one of the most common types of kidney cancer. While RCC tends to present as a localized tumor, a notable proportion may present with distant metastasis. In some instances, RCC may also present with intravascular tumor extension, often called tumor thrombus (TT). Its presence confers a worse prognosis and has important implications for the tumor's staging and treatment. Despite extensive documentation of RCC TT in the US, limited data exists regarding its presentation, management, and outcomes in Puerto Rico (PR). This study aims to broaden the available information on RCC TT, emphasizing surgical management and outcomes. We also provide descriptive data on patient demographics and clinical presentation to improve decision-making among clinicians caring for Puerto Rican men and women.

Methods: In this single-center, retrospective study, we evaluated patients who underwent partial or total nephrectomy at Saint Luke's Episcopal Medical Center between 2018 and 2022. Data was abstracted from electronic health records (EHR). Patients without documented evidence of TT during the peri-operative period were excluded from the study. A total of 220 patient records were evaluated, of which 12 met the inclusion criteria for the study. Cases were categorized using the latest RCC TT guidelines. Central tendency measurements were used to describe the sample distribution. The mean was considered to make assumptions regarding the prevalent observations, and the median was considered to rule out possible outliers. Categorical data were evaluated using proportion analyses, including TT extension level and BMI variables. Fisher’s exact test evaluated the association between the World Health Organization/International Society of Urological Pathology (WHO/ISUP) grade and TT extension level.

Results: Most patients lacked TT-related symptoms. The most severe presenting symptom was a pulmonary embolism (8.3%). Hypertension (83.3%), BMI greater than 25 at the time of diagnosis (75%), and type 2 diabetes mellitus (66.7%) were the most common comorbid conditions within our cohort. Nearly 75% of patients underwent laparoscopic radical nephrectomy with TT resection. One left-sided level III case was managed by laparoscopic-assisted open radical nephrectomy with a right subcostal incision. There were zero intraoperative complications and two postoperative complications. The histopathological reports of all cases were consistent with clear cell carcinoma, and half of the cases (n=6) were WHO/ISUP G4. All patients are alive and free of disease.

Conclusion: RCC is a common renal neoplasm in PR that can present with intravascular tumor extension. Our findings do not establish a definitive association between BMI, tumor size, WHO/ISUP grading, and TT extension level. Our study shows that laparoscopic removal of RCC TT is a safe and effective approach. However, the generalizability of our findings is limited by the study's design and sample size. Future research should focus on identifying predictive markers, establishing effective screening protocols, and determining if our hybrid approach has comparable outcomes to the standard open approach.

## Introduction

Renal cell carcinoma (RCC) is the most prevalent form of kidney cancer. As of 2020, RCC exhibited a global annual incidence of 400,000 new cases diagnosed and a 5-year survival rate of 77.6% [1-3]. Significant risk factors for RCC development include hypertension, obesity, dialysis in acquired cystic disease patients and smoking, contributing to a more advanced disease state [4-7]. Clear-cell carcinoma is the most common RCC subtype, accounting for 75% of all cases [8]. Most RCC cases present as localized neoplasms, although 30% of patients present with distant metastases in lymph nodes, lungs, liver, and bone [9]. While uncommon, RCC can occasionally manifest with intravascular tumor extension, known as a tumor thrombus (TT). The prevalence of RCC venous TT is approximately 13.3%, with renal vein involvement occurring in 10-18% of cases, inferior vena cava (IVC) involvement in 4-23% of cases, and right atrium involvement in 1% of cases [10-13]. Symptoms associated with RCC TT presentation include varicocele, lower extremity swelling, cardiac dysfunction, pulmonary embolism, or Budd-Chiari syndrome. However, in some instances, TT may remain asymptomatic and only be incidentally discovered through imaging [14-18]. Despite extensive documentation of RCC TT in the US, limited information exists regarding its presentation, management, and outcomes in Puerto Rico (PR). Therefore, this study aims to describe twelve RCC TT cases in the southern region of PR to investigate whether the presentation, management, and outcomes of RCC TT align with established literature. The study focuses on increasing the available information concerning this severe RCC complication to improve surgical management and survival outcomes in PR.

## Materials and methods

Patient selection

Approval from the Institutional Review Board (IRB) was obtained before the initiation of the study (IRB number: 2205104830) from the Ponce Research Institute. The initial inclusion criteria in our single-site retrospective study were patients who were > 21 years of age and had undergone partial or radical nephrectomy at St. Luke’s Episcopal Medical Center (Ponce, PR) from 2018 to 2022. This initial criterion identified a total of 220 patients. We then refined our inclusion criteria to only those with RCC, which required us to review each patient's pathological report to exclude other neoplasms such as sarcoma, oncocytoma, and urothelial carcinoma. This resulted in a cohort of 189. Subsequent refinement of our selection criteria, which involved excluding patients with localized kidney neoplasms and confirming intravascular extension through pathological reports and intra/post-operative notes, yielded a final cohort of 12 cases.

Surgical description

Cases classified as levels 0-II were managed through a standard laparoscopic approach. In Level 0 cases, vascular staplers were utilized for renal vein transection. Conversely, cases classified as level I and above necessitated thrombectomy and transection at the junction of the IVC and the renal vein, followed by primary closure of the venotomy.

Left-Sided Level III TT Without IVC Exploration

A chevron incision was employed to access the abdomen, and medial mobilization of the colon was conducted to expose the left kidney. The renal hilum was dissected, and the duodenum was then kocherized to access the IVC. Subsequently, the renal artery was transected, followed by the ureter, while the renal vein was preserved and freed from surrounding structures. After ensuring hemostasis, the vascular team joined the case for the dissection of the IVC.

The IVC TT was identified and moved (milked) cephalad into the renal vein. The IVC was then clamped caudally for proximal control, and a venotomy was performed in the renal vein at the junction with the IVC. The venotomy was primarily closed, and the specimen was removed en bloc and sent to pathology. 

Left-Sided Level III With IVC Exploration

Two 5 mm trocars were placed in the right subcostal margin at the level of the mid-clavicular line and anterior mid-axillary line, and a 10 mm trocar was placed in the left lower quadrant. The colon was reflected medially, and the kidney was dissected from surrounding structures. The ureter and gonadal vein were transected first, followed by the renal artery, with the renal vein preserved. At this juncture, the vascular surgery team joined the case for the dissection of the IVC.

The procedure was then converted to an open approach by connecting both subcostal trocar incisions. The steps to achieve proximal control of the IVC mirrored those described in the previous case. After securing proximal vascular control, a venotomy was performed in the renal vein at the junction with the IVC. Resistance was encountered while attempting to remove the TT, necessitating further exploration of the IVC. 

The IVC was dissected circumferentially up to the confluence of the hepatic veins, and the TT was found to extend into the supra-hepatic IVC. Vessel loops were placed around the infra-hepatic IVC and right renal vein, and the caudate lobe of the liver was mobilized. The TT was then moved (milked) caudally into the infra-hepatic IVC, and a vascular clamp was placed cephalad to achieve proximal control. Venous thrombectomy with a Fogarty catheter was performed through an incision in the IVC, which was then closed primarily. After ensuring hemostasis, the abdomen was closed in the standard manner.

Data collection

Demographic and clinical information was abstracted from the patient’s electronic health records. The Mayo Clinic RCC TT classification guideline (MCTTCG) was used to classify these patients according to the extent of intravascular tumor extension (Table 1) [19-21].

**Table 1:** Mayo clinic RCC tumor thrombus classification guidelines* ^*^The content of this table was extracted from the Mayo Clinic Tumor Thrombus Classification Guidelines and adapted for the article's use [21]. IVC: Inferior vena cava; RCC: Renal cell carcinoma

Tumor Thrombus Level	Description
0	Limited to the renal vein
l	Extends into the IVC, but < 2 cm above the renal vein
ll	Extends into the IVC, > 2 cm above the renal vein but below hepatic veins
lll	Extends above hepatic veins but below the diaphragm
lV	Extends above diaphragm

Statistical analysis

Central tendency measurements were used to describe the sample distribution. The mean was considered to make assumptions regarding the prevalent observations, and the median was considered to rule out possible outliers. Categorical data were evaluated using proportion analyses, including TT extension level and BMI variables. Variables with less than three events were excluded from the statistical test. Patients were stratified using their results from the BMI calculations. Individuals with a BMI of 18.5-24.9 were considered healthy in terms of weight. Patients with a BMI between 25.0 and 29.9 were classified as overweight, and patients with a BMI higher than 30 were considered obese [22]. Fisher's exact test was performed to assess the significance between the World Health Organization/International Society of Urological Pathology (WHO/ISUP) grade and TT extension level.

## Results

The study analyzed twelve patients: 58.3% (n=7) male and 41.7% (n=5) female. The mean and median age at the time of surgery were 63.6 (±6.4) and 63.0, respectively. As seen in (Table 2), 25% of patients were considered to have a healthy weight, and 75% were either overweight or obese. The most prevalent comorbidities in our cohort were hypertension (83.3%, n=10) and type 2 diabetes mellitus (66.7%, n=8). Additionally, hypercholesterolemia was observed in four patients (33%). Only one patient had a history of tobacco use. A positive family history of cancer was present in 25% (n=3) of patients. The most common presenting symptoms were flank pain (33.3%, n=4), hematuria (25%, n=3), and dysuria (16.7%, n=2) (Table 3). One patient presented to the emergency room with a pulmonary embolism (PE) (8.3%, n=1). None of the patients from our cohort presented with fever, cachexia, weight loss, palpable flank mass, lower limb edema, or right-sided varicocele.

**Table 2:** Patient demographics *Variable with missing value. RCC: Renal cell carcinoma

Demographics	RCC cases (n=12), n (%)
Sex	
Female	5 (41.7)
Male	7 (58.3)
BMI Categories	
<18.5	0 (0.0)
18.5-24.9	3 (25.0)
25.0-29.9	6 (50.0)
30.0-34.9	2 (16.7)
35.0-39.9	1 (8.3)
>40.0	0 (0.0)
Comorbidities	
Hypertension	10 (83.3)
Diabetes Mellitus II	8 (66.7)
History of Tobacco Use	1 (8.3)
Chronic Heart Disease	1 (8.3)
Family History of Cancer	
Yes	3 (25.0)
No	9 (75.0)
Age at surgery	
Mean ±SD	63.6 ±6.4
Median	63.0
Range (min-max)	53-73
BMI	
Mean ±SD	27.2 ±4.6
Median	26.7
Range (min-max)	19.8-36.0
Maximum Tumor Vol (cm³)*	
Mean ±SD	197.8 ±104.0
Median	215.3
Range (min-max)	38.4-324.4
Hospital LOS (days)	
Mean ±SD	1.0 ±2.2
Median	2.0
Range (min-max)	1.0-7.0

**Table 3:** Presenting symptoms RCC: Renal cell carcinoma

Presenting Symptoms	RCC cases (n%)
Flank Pain	4 (33.3)
Hematuria	3 (25.0)
Dysuria	2 (16.7)
Pulmonary Embolism	1 (8.3)

Patients were classified and grouped according to the level of TT extension based on the MCTTCG (Table 1). Initially, only eight patients had a TT on imaging, but this number increased to twelve upon surgical staging. As shown in Table 4, our cohort distribution based on the tumor thrombus level was as follows: Level 0 (n=6), level I (n=1), level II (n=2), level III (n=3), and level IV (n=0).

**Table 4:** Tumor thrombus level classification

Tumor Thrombus Level	Imaging Staging n (%)	Surgical Staging n (%)
0	3 (37.5)	6 (50.0)
l	0 (0)	1 (8.3)
ll	3 (37.5)	2 (16.7)
lll	2 (16.7)	3 (25.0)
lV	0 (0)	0 (0)
None Identified	4 (33.0)	0 (0)

The mean preoperative calcium, hematocrit, and platelet counts were 9.3 (±1.0), 39.1 (±6.8), and 297.8 (±121.0), respectively (Table 5). Two patients had hypocalcemia before surgery, and six were anemic (50%). No intraoperative complications were reported within the cohort under study; however, postoperative complications arose in two cases. The first case involved a level 0 patient who experienced vocal cord damage. The second case pertained to a right-sided level III patient who suffered an acute kidney injury. Transfusion volumes for level III patients were as follows: one left-sided level III case without IVC exploration received a transfusion of four units of packed red blood cells (PRBCs), each containing 300 mL and three units of plasma containing 250 mL. A right-sided level III case without IVC exploration involved a transfusion protocol comprising ten units of PRBCs, each 300 mL, and four units of plasma, each 300 mL. A left-sided level III case with IVC exploration necessitated seven units of PRBCs, each 300 mL, and five units of plasma, each 250 mL. It is important to note that the estimated blood loss volume was not assessed for our patient cohort.

**Table 5:** Preoperative laboratory findings RCC: Renal cell carcinoma

Preoperative Laboratory Findings	RCC cases (n=12)
Calcium (mg/dL)	
Mean ±SD	9.3 ±1.0
Range (min-max)	7.4-10.0
Hematocrit	
Mean ±SD	39.1 ±6.8
Range (min-max)	27.5-49.0
Platelets (x1000)	
Mean ±SD	297.8 ±121.0
Range (min-max)	125.0-499.0

The pathology reports on all 12 cases showed a diagnosis of clear-cell RCC, primarily present in the left kidney (58.3%, n=7). Most cases (50%, n=6) were WHO/ISUP grade 4. An association between WHO/ISUP grading and TT extension level was not seen (p=0.8182) (Table 6). As seen in (Table 2), the mean and median tumor volume were 197.8 (±104.0) cm^3^ and 215.3 cm^3^, respectively. One patient was not included in the tumor volume analysis due to missing information on this variable. Most of the cases (75%, n=9) were done in the standard fashion via laparoscopic radical nephrectomy (LRN) except three, which were done via open radical nephrectomy (ORN) (n=2) and laparoscopic-assisted open radical nephrectomy (LAORN) (n=1) (Table 7). Assistance from the vascular surgery team was necessary for three-level III cases. Of these, IVC exploration was required for one left-sided level III case. LRN cases (75%, n=9) utilized periumbilical incisions to remove specimens. A chevron incision was employed for access, and specimen extraction of a left-sided level III case. In contrast, a right-sided level III case utilized a right subcostal incision. The case done by LAORN was a left-sided level III and involved a right subcostal incision for access and specimen extraction. (Table 7).

**Table 6:** WHO/ISUP grading distribution with TT extension level ^*^p-value was calculated using a Fisher exact test. WHO/ISUP: World Health Organization/International Society of Urological Pathology; TT: tumor thrombus

	Tumor Thrombus Level				
WHO/ISUP	0	I	II	III	p-value*
G2	1	0	0	1	0.8182
G3	3	0	1	0	
G4	2	1	1	2	

**Table 7:** Surgical variables IVC: Inferior vena cava; RCC: Renal cell carcinoma

Surgical Variables	RCC cases (n=12), n (%)
Surgical approach	
Laparoscopic RN	9 (75.0)
Laparoscopic-Assisted Open RN	1 (8.3)
Open RN	2 (16.7)
Type of Surgery	
Nephrectomy without IVC exploration	11 (91.7)
Nephrectomy with IVC Exploration	1 (8.3)
Tumor Laterality	
Left	7 (58.3)
Right	5 (41.7)
Intra-operative complications	
Yes	0 (0)
No	12 (100.0)
Post-operative complications	
Yes	2 (16.7)
No	10 (83.0)
Distant Metastasis	
Yes	2 (16.7)
No	10 (83.3)
Specimen Removal Technique	
Chevron	1 (8.3)
Right subcostal	2 (16.7)
Periumbilical	9 (75.0)

**Figure 1:**
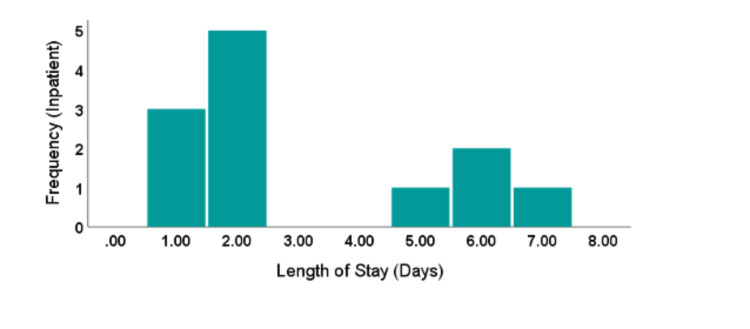
Distribution of patients by hospital length of stay Bars represent the frequency of the events based on a week’s time frame. The mode of stay was two days; no observation was obtained for 3 and 4 days, shifting the SD to 2.2 days of stay as an in-patient.

Distant metastasis was seen in two cases (16.7%), a level II and a level III TT. None of the patients had lymph nodes positive for malignancy (Table 7). Hospital length of stay (HLOS) showed a bimodal distribution (Figure 1), with a mean and median of 3.1 (±2.2) and 2.0 days, respectively (Table 2). The longest HLOS was seven days, seen in a right-sided level III patient, while the shortest HLOS was a single day in three level 0 patients (Table 2).

## Discussion

Renal cell carcinoma can occasionally manifest with intravascular tumor extension, known as a tumor thrombus (TT). Despite the limited amount of molecular-level information on this progression, it is believed to be directly related to tumor advancement, being an intraluminal extension. However, some suggest that the expression of vascular endothelial growth factor, an essential element for RCC growth, does not play a role in the pathogenesis of tumor venous extension [23]. Furthermore, Shi Y. et al. unveiled the critical role of extracellular matrix (ECM) remodeling in the growth of tumor thrombus, thus implying the potential therapeutic utility of agents targeting ECM growth factors [24]. Additionally, recent research has identified genetic variations among patients with inferior vena cava/renal vein TT with and without vascular wall invasion. Notably, individuals lacking wall invasion exhibited a higher prevalence of mutations in PTEN, TP53, various epigenetic regulators, and elevated expression of cell cycle-related pathway genes [25]. These findings allude to an intricate yet structured pathway underlying TT development, a phenomenon that, despite recent discoveries, still needs to be outlined at the molecular level. Nonetheless, it is worth highlighting that the clinical aspects of this ailment are well-documented.

According to the literature, the presentation of TT in RCC patients is variable; some remain asymptomatic, while others exhibit symptoms like varicocele, lower extremity swelling, cardiac dysfunction, pulmonary embolism, or Budd-Chiari Syndrome [14-18]. In our patient cohort, as shown in Table 3, none displayed symptoms indicative of a TT, except for one case of pulmonary embolism (PE). This patient presented to the emergency room with signs and symptoms of PE. Immediate anticoagulation therapy was administered, and subsequent CT angiography revealed a bland pulmonary embolus and a level II TT. The PE was managed pharmacologically, and the patient was scheduled for elective resection of the TT and kidney. The occurrence of PE did not alter the outcome of the surgery; however, it emphasizes the need for further research into identifying patients with RCC who are at an elevated risk of experiencing severe complications related to PE.

Obesity, diabetes, and hypertension have been well associated with renal neoplasms. These are highly prevalent in PR, with a prevalence of 68% for obese and overweight individuals, 15.8% for diabetes, and 41% for hypertension [6, 26-29]. Specifically, obesity has been known to promote and further the development of RCC. The mechanism by which it furthers tumorigenesis is thought to be due to excess adipose tissue, which promotes the secretion of adipokines, pro-inflammatory and pro-angiogenic factors, and serves as an energy reservoir for the tumor [6]. Similarly, hypertension induces a state of chronic inflammation, promoting hypoxia-induced factor expression with subsequent cell growth dysregulation and angiogenesis. Additionally, hypertension is associated with increased formation of reactive oxygen species, which promotes tumor development and progression [24]. Diabetes, on the other hand, has been associated with worse overall survival, cancer-specific survival, and recurrence-free survival [27]. In our patient group, as depicted in Table 2, diabetes, a BMI ≥ 25, and hypertension were highly prevalent (66.7%, 75%, and 83.3%, respectively). As previously mentioned, the progression of the TT depends on factors that promote accelerated tumor growth, such as hypoxia, inflammation, insulin resistance, excessive adipose tissue, and adipokines [6]. Our data does not support such a relationship due to the sample size; however, most of the patients were either overweight or obese.

As shown in Table 4, four patients had no evidence of tumor extension into the renal venous vasculature on pre-operative imaging. Some of these patients had large tumors, which one would typically expect to exhibit vascular extension on pre-operative imaging. These extensions were discovered during surgical exploration and pathological assessment of the specimens. One might assume that regional structures would have greater involvement as tumor size increases, but our data does not support this assumption. 

The primary surgical approach in the case series was laparoscopic radical nephrectomy with periumbilical specimen removal (Table 7). While effective, this minimally invasive approach is generally reserved for lower-level TT. For those with greater extension, an open surgical approach is often necessitated. In our effort to explore alternative methods, we implemented a new technique involving the laparoscopic dissection of a left-sided kidney using ports positioned on the right side, followed by a connection of the trocar incisions to form a right subcostal incision. This approach leverages the anatomical proximity of the IVC to the right flank. The technique potentially offers a less burdensome approach for patients, mitigating the overall operative impact.

Regardless of the approach, TT extension level, or laterality, none of our patients experienced intraoperative complications. This is interesting, considering that most (58.3%, n=7) of our patients had left-sided tumors (Table 7), which typically require a more challenging procedure and present with a more aggressive nature [29]. Our findings align with Zhang et al.'s assertion that laparoscopic removal of RCC TT is challenging yet safe and feasible [30]. Nevertheless, we did have two postoperative complications: one who developed vocal cord damage leading to hoarseness and respiratory difficulties secondary to atelectasis and edema and one who developed acute kidney injury secondary to hypovolemia. All noted complications were successfully managed, permitting a complete post-operative recovery in all patients. Despite the technical complexity of these cases, all patients are alive. Nine have been cured, while three level III are in remission. These three are being treated with pembrolizumab as a preventive measure based on the high-risk staging determined at their initial presentation.

The hospital length of stay (HLOS) for our patient group, as seen in Table 2, was predominantly ≤2 days (66%), primarily for those with a TT extension level of I or II. This is unsurprising, given that patients with higher TT extension levels typically require more prolonged and invasive procedures, potentially increasing the patient burden. It is worth noting that Rabinowitz’s study reported a median HLOS of 7 days for cases involving the IVC. However, comparing our results directly is challenging, as most of their cases were open procedures, not laparoscopic [8]. Additionally, their study only included those with IVC extension, whereas ours also encompassed cases with localized renal vein extension in addition to IVC.

Pathological assessment revealed that all our patients had clear cell carcinoma, consistent with the literature, which asserts that clear cells are primarily responsible for the development of TT. Intriguingly, in our cohort, no significant association was observed between WHO/ISUP grading and the level of TT extension, suggesting the potential for other contributing factors in TT pathogenesis and progression (Table 6). It is worth noting that other RCC variants may also cause TT, and literature suggests that perioperative and oncologic outcomes of clear-cell and non-clear-cell TT are comparable [8]. However, this observation does not diminish the need for further research, especially in the Hispanic population, where an increased risk for obesity is associated with a worse disease-specific survival in RCC [31].

In PR, TT clinical and pathological information reporting to the Cancer Registry is not mandatory, limiting our ability to track the incidence, prevalence, and mortality associated with RCC. This hinders our ability to gauge the condition’s relevance and the risk of life-threatening complications within our population. One obstacle to fully understanding the disease's manifestation is the reliance on primary care physicians (PCP) to recognize patients with high-risk renal tumors and promptly refer them to a urologist for TT evaluation. To address this issue, it is essential to equip PCPs with the latest screening guidelines, ensuring patients displaying risk factors or indicative symptoms are promptly identified and receive timely referrals to urologists for evaluation.

## Conclusions

Renal cell carcinoma is a common renal neoplasm in PR, with the propensity to progress into the renal vasculature. Our findings failed to establish a definitive association between BMI, tumor size, and TT extension level. Additionally, there was no relationship between WHO/ISUP grading and TT extension level. Our results indicate that the laparoscopic excision of RCC-associated TT is a viable and effective alternative to conventional open methods, as evidenced by the current disease-free status of all 12 patients. Moreover, we propose that a hybrid technique, integrating laparoscopic and open surgical approaches for the excision of higher-level left-sided TT, represents a plausible innovative strategy.

Further studies are necessary to evaluate whether this hybrid approach yields outcomes equivalent to those of traditional open surgery. The study's design and small sample size limit our findings' generalizability, underlining the need for broader research, especially within Hispanic populations, to better understand the underlying factors that influence the development of RCC TT. Additionally, identifying predictive markers could significantly enhance clinicians' capacity to detect and manage patients at a greater risk for life-threatening TT complications.
